# One year outcomes of intravitreal faricimab for treatment Naïve neovascular AMD and associations with baseline aqueous humor cytokines

**DOI:** 10.1038/s41598-025-28911-9

**Published:** 2025-12-29

**Authors:** Satoru Inoda, Hidenori Takahashi, Yuto Hashimoto, Hana Yoshida, Hironori Takahashi, Takuya Takayama, Shouma Tsuchiya, Daizo Matsumoto, Hidetoshi Kawashima, Toshikatsu Kaburaki, Yasuo Yanagi

**Affiliations:** 1 Department of Ophthalmology, Jichi Medical University, 3311-1 Yakushiji, Shimotsuke-shi, Tochigi 329-0498 Japan; 2 Center for Cyber Medicine Research, University of Tsukuba, Tsukuba-shi, Ibaraki Japan; 3 Department of Ophthalmology and Micro-Technology, Yokohama City University, Yokohama, Japan; 4 Retina Research Group, Singapore Eye Research Institute, Singapore Eye-ACP, Duke-NUS Medical School, National University of Singapore, Singapore, Singapore

**Keywords:** Age-related macular degeneration, Faricimab, Ang-1, Diseases, Eye diseases

## Abstract

This study investigated the one-year real-world clinical outcomes of intravitreal faricimab (IVF) in treatment-naïve neovascular age-related macular degeneration (nAMD) and its association with aqueous humor cytokine profiles. Thirty-four eyes of 32 nAMD patients, who received initial IVF at Jichi Medical University were included. These eyes showed significant improvements in central retinal subfield thickness (CST), central choroidal thickness (CCT), and best-corrected visual acuity (BCVA) one year post-IVF (all *P* < 0.01). Among 24 measurable cytokines, only VEGF-A was significantly higher in eyes with a dry macula at week 16 (*P* = 0.0030). Three cytokines were significantly higher in eyes with longer injection interval. Higher levels of galectin-1 and VCAM-1, and lower levels of P-selectin, were correlated with greater CST reduction (*P* = 0.020, 0.0061, and 0.012, respectively). Conversely, five cytokines including Ang-1 were correlated with greater BCVA improvement (all *P* < 0.05). In conclusion, an anatomical perspective faricimab demonstrated anatomical efficacy for VEGF-driven nAMD in the loading phase, while multiple cytokines associated with vascular instability or fibrosis appear to contribute to its durability. Notably, Ang-1 may be linked to visual improvement, suggesting that the neurotrophic effects of Ang-1 could be enhanced by Ang-2 inhibition.

**Supplementary Information:**

The online version contains supplementary material available at 10.1038/s41598-025-28911-9.

## Introduction

Four anti-vascular endothelial growth factor (VEGF) drugs are currently approved for neovascular age-related macular degeneration (nAMD): ranibizumab, aflibercept, brolucizumab, and faricimab. Each of these agents has shown high efficacy and distinct properties due to their small molecular weights and/or elevated affinity for VEGF. Whereas the first three agents specifically target the VEGF family, faricimab blocks both angiopoietin (Ang)-2 and VEGF.

The TENAYA and LUCERNE trials showed a prolonged effect of faricimab compared with aflibercept in terms of maintaining best corrected visual acuity (BCVA) outcomes. In subanalyses of those trials that specifically considered ethnicity and some short-term outcomes in Asians, faricimab appeared to exhibit superior efficacy in individuals of Asian descent^1–4^. Regarding the efficacy of faricimab in previously treated individuals, a previous report from Japan suggested that intravitreal faricimab (IVF) has a favorable safety profile and satisfactory effectiveness that make it a promising alternative to brolucizumab in the treatment of nAMD^5,6^. These reports showed the particularly favorable results of faricimab in Asian patients with nAMD. This improved efficacy could be due to racial differences but might also be due to factors such as differences in the proportions of macular neovascularization (MNV) disease types. Pachychoroid-associated MNV is more common in Asians, and a previous report noted that there was no correlation between VEGF and Ang-2 concentrations and that pachychoroid-associated MNV was associated with elevated Ang-2^7^.

Previous studies have investigated the correlation between anatomical outcomes and visual function outcomes, but the conclusion has not simply been that a “dry” retina is always better^8–11^. Nonetheless, some correlations between anatomy and visual prognosis have been elucidated. Dryness at 16 weeks after the initiation of anti-VEGF therapy has been associated with subsequent treatment responsiveness^12–14^ and is gaining attention as a useful factor for selecting personalized treatment in an era of multiple drug options. Furthermore, fibrosis has been linked to poor visual prognosis, and faricimab is expected to have a fibrosis-suppressing effect^15,16^. In addition to the association between anatomical and visual outcomes, cytokine profiling in nAMD is another crucial research area. For instance, previous studies have demonstrated elevated levels of interferon-gamma-inducible protein 10 (IP-10), interleukin-10 (IL-10), and interferon-gamma (IFN-γ) in the eyes of nAMD patients compared to controls. These cytokines have been associated with central subfield thickness (CST), the response to anti-VEGF agents, and the progression of AMD^17–21^.

This study aims to assess how baseline intraocular cytokine levels influence anatomical outcomes, treatment response, and prognosis in treatment-naïve nAMD patients receiving faricimab. Understanding these associations may provide insights into personalized treatment strategies and faricimab’s mechanisms beyond VEGF inhibition.

## Methods

### Ethical approval and consent

This study had a single-center retrospective design and was approved by the institutional review board of Jichi Medical University (JICHI 23 − 009) and adhered to the tenets of the 1964 Declaration of Helsinki and its later amendments or comparable ethical standards. The study procedures followed our institutional guidelines, and all patients provided written informed consent before the procedures were performed. The study was registered in the UMIN Clinical Trials Registry (http://www.umin.ac.jp/) under the unique trial number UMIN000020718 (date of registration: October 31, 2024).

### Procedure

A total of 39 eyes of 37 consecutive patients with treatment-naïve nAMD treated with their first three consecutive IVF injections between June 2022 and October 2022 were enrolled in this study. These patients received their first IVF injection at Jichi Medical University, and all were followed up for 1 year. Patients were excluded if they had myopia exceeding − 6 diopters, a history of uveitis, or a history of vitrectomy. Additionally, 2 eyes from 3 cases were excluded due to AMD onset more than 6 months prior to the study or an unclear onset of vision loss. Five eyes and 5 patients were excluded for the following reasons: 2 were lost to follow-up and 3 switched to brolucizumab. Therefore, this study included 34 eyes of 32 patients.

MNV was diagnosed based on fundus examination with multimodal imaging: fundus photography, fluorescein angiography and indocyanine green angiography (Heidelberg Retina Angiography; Heidelberg Engineering, Heidelberg, Germany), or swept-source optical coherence tomography (OCT, DRI OCT Triton; Topcon, Tokyo, Japan). Color fundus photographs were obtained using a commercially available fundus camera system (VX-10; Kowa Co., Ltd., Nagoya, Japan). Patients are diagnosed with nAMD according to the following classification criteria: (1) choroidal neovascularization, (2) serous pigment epithelial detachment, (3) hemorrhagic pigment epithelial detachment, and/or (4) fibrotic scarring^22^. This classification is based on the 2008 Japanese guidelines for nAMD at the time, and multiple studies have referenced these guidelines^7,23–25^. Additionally, pachychoroid features were diagnosed using multimodal imaging analysis, which include the presence of choroidal vascular hyperpermeability detected in the late phase of indocyanine green angiography, dilated choroidal vessels below type 1 MNV detected by indocyanine green angiography or OCT, and the presence of central serous chorioretinopathy or pachychoroid pigmented epitheliopathy related to retinal pigment epithelium abnormalities^26–28^. Pachychoroid features were defined carefully with attention to inner choroidal attenuation in addition to those features.

Approximately 0.2 mL aqueous humor (AqH) was collected using a 30-G needle immediately before the IVF injection from patients with nAMD. The sample was immediately transferred to a sterile tube and stored at − 80 °C until analysis.

### Treatment regimen

Patients received monthly injections for 3 consecutive months and were then treated on a modified treat-and-extend regimen described in the section below. Briefly, in this regimen, the injection interval was extended in 4-week increments until the initial occurrence of exudative changes. Then, the interval was reduced in 2-week decrements, and disease activity was defined as new retinal hemorrhage, presence of fluid on OCT, or a change in the pigmented retinal detachment. With swept-source OCT, 9-mm radial scans through the foveal center were performed. Fluid was defined as the presence of any intraretinal fluid and any subretinal fluid greater than 100 μm in height at the subfoveal center. Subfoveal subretinal fluid of 100 μm or less or any subretinal fluid elsewhere was tolerated if it did not affect vision, and the treatment period was maintained under these conditions^9–11^.

#### STEP-C protocol [Structured Treat and Extend, and PRN with controlled monitoring protocol (formerly referred to as mTAE)]

The patient underwent treatment with a “modified TAE” procedure, including a 3-cycle monthly induction phase. We refer to this modified TAE procedure as the STEP-C Protocol. STEP is an acronym derived from Structured Treat and Extend, and PRN with controlled monitoring. This protocol aims for a planned transition approach from active treatment to maintenance management with monitoring. To ensure optimal disease control while minimizing overtreatment, it establishes three steps: Step-A: Induction Phase, Step-B: Maintenance Phase, and Step-C: PRN with controlled monitoring. Each step involves management with defined achievement goals. Step-A, the induction phase, aims for the maximum functional and anatomical outcome, consisting of (at least) three consecutive monthly injections with OCT examinations and clinical evaluations. If activity persists, additional treatment is administered before transitioning to Step-B. The achievement criterion for Step-A is the resolution (inactivity) or stabilization of exudative changes (i.e., intraretinal or subretinal fluid as revealed by OCT) in the central subfield.

Step-B aims to stabilize the maximum effect achieved in Step-A while increasing treatment intervals. The intravitreal injection interval ranges from a minimum of every 8 weeks to a maximum of every 16 weeks. The initial treatment interval is 8 weeks, and if there are no exudative changes, the interval is extended every 4 weeks. Injections are also administered to maintain the no retinal fluid, but if recurrence occurs, the treatment interval is shortened, and after the first recurrence, the interval is adjusted every 2 weeks. Note that we believe that this adjustment protocol could be modified according to the physicians’ discretions and depending the drugs used. The achievement criterion for Step-B is achieving stability with a 16-week treatment interval. Transition to Step-C can happen after achieving dry macula twice with a 16-week interval after discussing the potential risks and benefits with the patients. Following achievement of Step-C, observation is continued, transitioning from prophylactic injections to PRN.

### Outcome measures

As anatomical outcomes, we analyzed factors associated with a dry macula at 16 weeks, the treatment interval at 1 year, and changes in CST and central choroidal thickness (CCT). As a functional outcome, we analyzed factors associated with changes in BCVA.

Visual acuity was measured using decimal visual acuity, which was then converted to logMAR units for outcome analyses. CST and CCT were measured at the foveal center using OCT, from Bruch’s membrane to the inner sclera and from then retinal pigment epithelium (RPE) outer edge to the inner sclera, respectively.

Changes in CST, CCT, BCVA, and dry macula at 16 weeks were compared to baseline cytokine levels measured over 1 year. To assess the impact of the treatment interval on 1-year outcomes, patients were divided into two groups based on injection frequency: a long-interval group and a short-interval group. After adjusting for age, sex, and axial length, baseline cytokine levels were compared between the two groups.

In a safety outcome evaluation, we assessed potential adverse effects such as intraocular inflammation, retinal vasculitis, RPE tear, and severe vision loss (15 letters or more) due to any cause and procedure-related adverse effects such as traumatic cataract formation and retinal tear, as well as any systemic adverse effects reported by the patient.

### Multiplex cytokine assay

The concentrations of the following cytokines were analyzed in AqH samples collected between November 2023 and December 2023 using Bio-Plex-Pro™ Human Chemokine Assays (Bio-Rad, Hercules, CA) according to the manufacturer’s instructions: C-X-C motif chemokine ligand 1 (CXCL1), CXCL11, CXCL13, IP-10, C-C motif chemokine ligand 2 (CCL2), CCL7, CCL11, IL-1α, IL-1β, IL-2, IL-5, IL-6, IL-8, IL-10, IL-12p, IL-17, tumor necrosis factor-alpha (TNF-α), IFN-γ, granulocyte colony-stimulating factor (G-CSF), macrophage colony-stimulating factor (M-CSF), granulocyte macrophage colony-stimulating factor (GM-CSF), intercellular adhesion molecule-1 (ICAM-1), vascular cell adhesion molecule-1 (VCAM-1), P-selectin, galectin-1, matrix metallopeptidase 1 (MMP-1), MMP-9, Ang-1, Ang-2, platelet-derived growth factor-AA (PDGF-AA), PDGF-AB, PDGF-BB, placental growth factor, and VEGF-A. The detection limits (in pg/mL) were 49.8, 24.7, 18.5, 2.67, 1.65, 30.9, 61.7, 4.57, 17.7, 4.94, 30.9, 6.17, 3.25, 4.12, 4.1, 144, 13.2, 8.23, 2.67, 24.7, 103, 12.3, 3200, 8850, 206, 1750, 49.4, 123, 94.7, 94.7, 4.94, 25.4, 12.3, 2.88, and 11.5, respectively.

We measured undiluted AqH samples. Measurements were performed twice for each sample and the average was calculated and used for the analysis. Cytokine concentrations beyond the standard curve were extrapolated. For values below the extrapolation limit, half of the lowest quantifiable concentration was used^29^.

### Statistical analysis

Categorical data were assessed using Pearson’s chi-square test or Fisher’s direct probability test. After confirming a normal distribution using the Shapiro–Wilk test, continuous variables were evaluated using a Student’s *t*-test, Wilcoxon rank sum test, and one-way analysis of variance (ANOVA). Multiple regression analysis was used to evaluate associations between each cytokine and changes in BCVA, CST, and CCT, the intended injection interval at the last visit, and dryness at 16 weeks after the first injection. To assess the robustness of our findings, we performed a bootstrap analysis with 1000 resamples. A mediation analysis was performed to examine direct and indirect relationships between changes in BCVA, CST, and CCT and associated cytokines that were revealed by multiple regression analysis. Repeat tests were not performed, so no correction was applied for multiple comparisons.

All statistical analyses were performed using JMP Pro software ver. 17.0.0 (SAS Institute Inc., Cary, NC) and jamovi, ver. 2.5.6.0. A *P* value of < 0.05 was considered statistically significant. Results are reported as the mean ± standard deviation (SD).

## Results

### Clinical outcomes

Table 1 shows the patients’ characteristics in this study. Mean age was 75.7 ± 8.55 and there were 22 men (69%). BCVA, CST, and CCT significantly improved over 1 year after the first IVF injection compared with baseline (all *P* < 0.001) (Table 2, See Supplementary Fig. 1). There were no significant associations of changes in BCVA, CST, and CCT and the injection interval at the last visit with baseline demographic factors (including age, axial length, sex, any exudative changes during the induction phase, and pachychoroid/drusen features).

**Table 1:** Patient characteristics.

	nAMD
N (eye)	34 (32)
Age, years	75.2 ± 8.48
Sex, male (%)	22 (69)
Axial length, mm	23.71 ± 0.96
BCVA, logMAR	0.479 ± 0.519
CST, µm	311 ± 68.9
CCT, µm	191 ± 103.0
Disease subtype*, [eyes]	14, 4, 6, 10

*Macular neovascularization (MNV) type 1, MNV type 2, MNV type 3, and polypoidal choroidal vasculopathy.

AMD, age-related macular degeneration; BCVA, best corrected visual acuity; CST, central subfield retinal thickness; CCT, central choroidal thickness.

**Table 2:** Changes in BCVA, CST, and CCT.

	Baseline *N* = 34	12 months after the first IVF *N* = 32*	*P* value^†^
BCVA, logMAR	0.479 ± 0.519	0.336 ± 0.539	**< 0.001**
CST, µm	311 ± 68.9	229 ± 47.5	**< 0.001**
CCT, µm	191 ± 103.0	170 ± 93.7	**< 0.001**

*One case of intraocular inflammation occurred, and the other developed endophthalmitis.

†Paired *t*-test.

BCVA, best corrected visual acuity; CST, central subfield retinal thickness; CCT, central choroidal thickness; IVF, intravitreal faricimab.

**Fig. 1:**
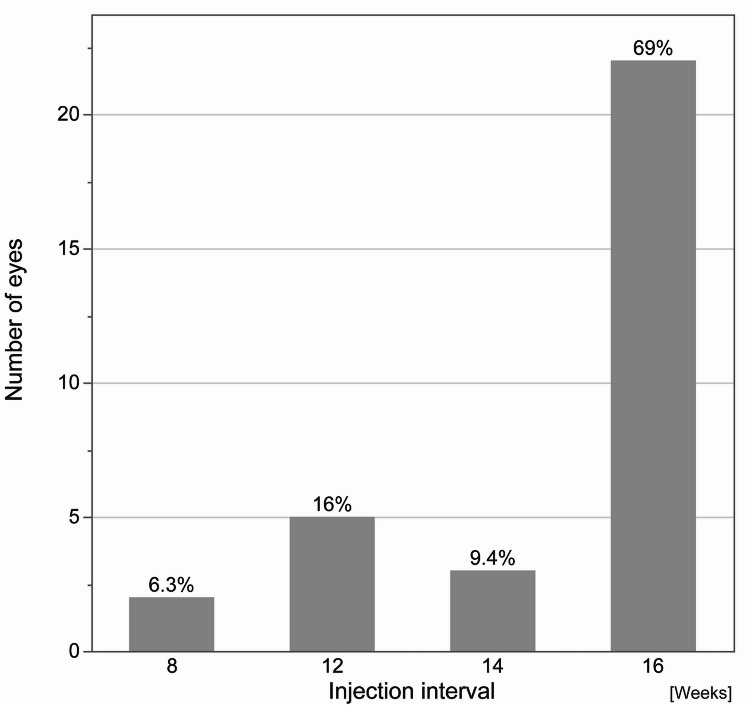
Intended injection intervals at the last visit. Almost 95% of patients maintained a treatment interval of over 12 weeks, while 6.3% of patients needed injections every 8 weeks.

**Fig. 2:**
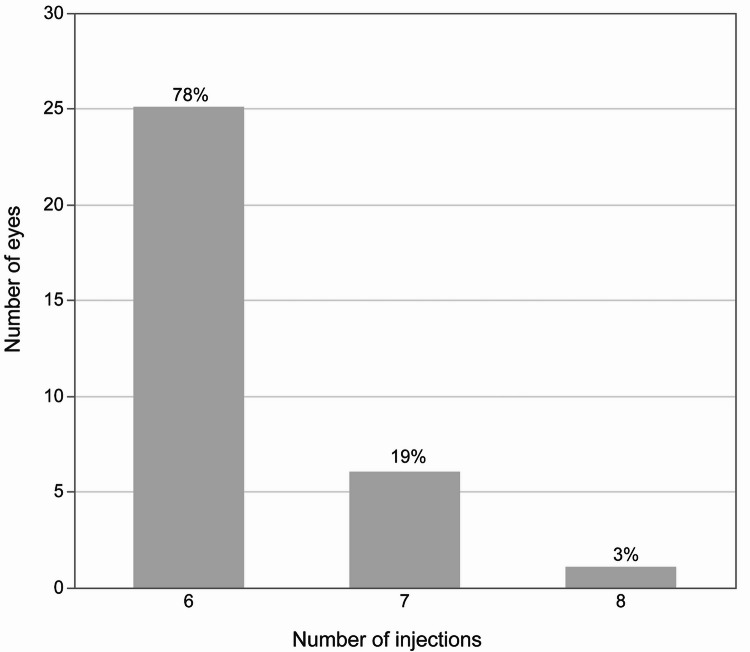
Number of injections. The mean number of injections was 6.1 ± 0.7.

More than 90% of the injection interval at the last visit were greater than 12 weeks (Fig. 1), with a mean number of injections being 6.1 ± 0.7. (Fig. 2). The total number of injections was not significantly different between individuals who achieved a dry macula (those with no IRF and SRF) at week 16 and those that did not (*P* = 0.14).

### Cytokine profiles

We measured the 34 cytokines mentioned in the Methods section; 10 cytokines were below the detection sensitivity: CXCL1, CXCL11, IL-1α, IL-1β, IL-5, IL-12-p70, IL-17, CCL7, G-CSF, and ICAM-1. Thus, we included the 24 cytokines that could be reliably measured.

### Association with anatomical outcomes

As anatomical outcomes, we analyzed factors associated with a dry macula at 16 weeks, the treatment interval at 1 year, and changes in CST and CCT. VEGF-A was significantly higher in patients with a dry macula at 16 weeks post-initial IVF (odds ratio (OR) = 11, *P* = 0.030) (Table 3, See Supplementary Fig. 2) Area under the receiver operating characteristic curve analysis showed area under the curves was 0.81 with a sensitivity of 67% and specificity of 100%. (See Supplementary Fig 0.3). At 1 year post-initial IVF injection, IL-10, IFN-γ, GM-CSF, and P-selectin were significantly elevated in patients with 16-week injection intervals compared to those with 12- or 8-week intervals. (OR = 23, 2.5*10^2^, 1.5*10^2^, and 4.8*10^2^, *P* = 0.0015, 0.031, 0.014, and 0.019, respectively) (Table 3, See Supplementary Fig. 4). Area under the receiver operating characteristic curve analysis showed area under the curves was 0.88 with a sensitivity of 86% and specificity of 100%. (See Supplementary Fig. 5)

**Table 3:** Association of cytokines with dryness at 16 weeks after the first injection, injection interval 12 months after the first IVF, and changes in BCVA, CST, and CCT after adjustment for each baseline Value, Age, Sex, and axis Length.

	Changes in BCVA		Changes in CST		Changes in CCT		Dryness at 16 Weeks After the First Injection		Injection intervals 12 months after the first IVF (16 weeks)	
	Estimate (95% CI)	P value	Estimate (95% CI)	P value	Estimate (95% CI)	P value	Estimate (95% CI)	P value	Estimate (95% CI)	P value
Angiopoietin-1	0.19 (8.9*10^− 3^ – 0.78)	**0.045**	43 (− 15–1.0 *10^2^)	0.14	− 13 (− 60–35)	0.59	4.7 (0.036–8.1*10^3^)	0.57	8.6 (0.030–2.3 *10^4^)	0.48
IL-6	− 6.3*10^− 3^ (− 0.067–0.054)	0.83	3.0 (− 6.0–12)	0.49	0.05 (− 6.6–6.7)	0.99	1.4 (0.72–2.7)	0.29	0.88 (0.31–1.9)	0.75
IL-8	0.69 (− 0.27–0.41)	0.68	40 (− 9.2–90)	0.11	7.3 (− 31–45)	0.70	0.12 (1.9*10^− 3^ – 5.9)	0.26	1.4 (0.032–1.0 *10^2^)	0.87
MCP-1	0.23 (− 0.26–0.73)	0.35	60 (− 11–1.3*10^2^)	0.09	13 (− 43–69)	0.63	0.13 (4.8*10^− 4^ – 72)	0.49	0.30 (1.2*10^− 3^ – 50)	0.63
TNF-α	0.052 (− 0.050–0.15)	0.31	8.8 (− 6.0–24)	0.23	− 8.3 (− 20–3.9)	0.17	0.87 (0.21–2.9)	0.83	2.0 (0.56 − 8.1)	0.29
Angiopoietin-2	0.22 (− 0.10–0.54)	0.17	22 (− 26–70)	0.35	− 21 (− 60–18)	0.28	2.4 (0.053 − 2.3*10^2^)	0.67	16 (0.14–7.3 *10^3^)	0.28
VEGF-A	2.6*10^− 3^ (− 0.21–0.21)	0.98	− 20 (− 50–40)	0.19	− 14 (− 38–9.8)	0.24	11 (1.3–2.2*10^2^)	**0.030**	2.0 (0.17 − 27)	0.54
PlGF	6.8*10^− 3^ (− 0.47–0.49)	0.98	28 (− 42–97)	0.42	− 22 (− 78–33)	0.42	28 (0.056–1.0*10^5^)	0.32	1.9 (0.024 − 2.7 *10^3^)	0.56
IP-10	0.19 (0.049–0.33)	**0.010**	− 3.3 (− 25 − 19)	0.76	13 (− 7.5–33)	0.21	0.79 (0.15–5.7)	0.80	2.2 (0.43 − 22)	0.39
IL-2	− 0.040 (− 0.37–0.29)	0.81	27 (− 21–74)	0.26	− 18 (− 56–21)	0.35	4.7 (0.076–3.0*10^3^)	0.52	48 (0.33 − 4.4 *10^4^)	0.19
IL− 10	0.18 (0.026–0.33)	**0.023**	− 0.32 (− 24–23)	0.98	− 8.8 (− 30–12)	0.40	1.2 (0.19 − 11)	0.85	23 (1.6 − 1.2 *10^3^)	**0.015**
IFN-γ	− 0.083 (− 0.39–0.22)	0.58	19 (− 26–63)	0.40	− 22 (− 57 − 13)	0.20	7.3 (0.14 − 2.0 *10^3^)	0.37	2.5*10^2^ (1.6–4.4 *10^5^)	**0.031**
GM-CSF	0.20 (0.021–0.38)	**0.030**	2.6 (− 25–31)	0.84	− 3.4 (-29–22)	0.78	0.78 (0.099 − 10)	0.82	1.5*10^2^ (20–1.5*10^5^)	**0.014**
M-CSF	0.035 (− 0.029–0.099)	0.27	3.9 (− 5.7–14)	0.41	2.2 (− 5.3–9.6)	0.56	0.25 (1.1*10^− 3^ – 1.4)	0.25	0.93 (0.16–1.9)	0.88
PDGF-AA	0.16 (− 0.29–0.61)	0.48	28 (− 39 − 94)	0.40	− 9.1 (− 62–45)	0.73	1.0 (2.6*10^− 3^ − 2.9 *10^2^)	0.99	3.7*10^− 3^ (1.3 *10*^−6^ − 1.1)	0.054
PDGF-AB	− 0.038 (− 0.34–0.26)	0.80	− 29 (− 71–14)	0.18	− 7.5 (− 42–27)	0.66	0.068 (5.2*10^− 4^ – 3.3)	0.18	8.2 (0.17 − 8.1 *10^2^)	0.29
PDGF-BB	0.090 (− 0.28–0.46)	0.62	15 (− 39–70)	0.57	− 23 (− 69–23)	0.31	40 (0.26 − 3.5 *10^4^)	0.16	1.6*10^2^ (0.95 1.9*10^5^)	0.090
VCAM-1	0.31 (− 0.12–0.73)	0.15	81 (25–1.4*10^2^)	**0.0061**	− 0.52 (− 50–50)	0.98	0.098 (4.2*10^− 4^–19)	0.37	0.05 (1.3*10^− 4^ – 6.6)	0.24
CXCL-13	0.26 (0.026–0.50)	**0.031**	16 (− 19–51)	0.35	3.6 (− 25–32)	0.8	0.18 (2.8*10^− 3^ − 1.4)	0.80	2.2 (0.18–50)	0.54
MMP-1	0.057 (− 0.26–0.37)	0.71	36 (− 7.5–80)	0.10	0.40 (− 37–38)	0.98	0.19 (4.4*10^− 3^ − 7.3)	0.36	7.1 (0.16–1.1*10^3^)	0.33
MMP-9	0.0067 (− 0.065–0.078)	0.85	4.1 (− 6.1–14)	0.42	− 3.9 (− 12–4.0)	0.32	0.18 (2.8*10^− 3^ − 1.4)	0.18	0.85 (0.25 − 1.7)	0.67
Galectin-1	0.31 (− 0.079–0.69)	0.11	63 (11–1.2*10^2^)	**0.020**	− 6.8 (− 54–40)	0.77	0.0072 (5.2*10^− 5^ – 7.4)	0.26	0.52 (4.8*10^− 3^ -71)	0.78
E-selectin	0.16 (− 0.39–0.72)	0.55	24 ( − 56–1.1*10^2^)	0.54	− 4.5 ( − 74–65)	0.90	0.055 (2.0*10*^−5^ − 55)	0.42	1.3*10^2^ (0.22 − 3.7*10^5^)	0.14
P-selectin	− 0.057 (− 0.38–0.27)	0.72	− 58 (− 1.0*10^2^ – − 13)	**0.012**	7.3 (− 30–44)	0.69	0.069 (2.1 *10^− 4^–5.5)	0.24	4.8*10^2^ (2.3 − 3.2 *10^6^)	**0.019**

BCVA, best corrected visual acuity; CST, central subfield retinal thickness; CCT, central choroidal thickness; IVF, intravitreal faricimab; CI, confidence interval; OR, odds ratio.

Higher levels of galectin-1 and VCAM-1 and a lower level of P-selectin were significantly associated with a greater reduction in CST. (*P* = 0.020, 0.0061, and 0.012) (See Supplementary Fig. 6). Bootstrap analysis (1000 resamples) yielded similar p-values for these associations, supporting the robustness of our findings. (See, Supplementary Table S1)

### Association with functional outcome

As a functional outcome, we analyzed factors associated with changes in BCVA, which revealed that lower levels of Ang-1, IP-10, IL-10, GM-CSF, and CXCL-13 were significantly associated with better changes in BCVA (*P* = 0.045, 0.010. 0.023 0.030, and 0.031) (See Supplementary Fig. 7). Bootstrap analysis (1000 resamples) yielded similar p-values for these associations, supporting the robustness of our findings. (See, Supplementary Table S1) To exclude the possibility that these effects were mediated by other factors, such as an anatomical response, mediation analysis was performed. The results indicated a total relationship between the change in BCVA and Ang-1 and a direct relationship of the change in BCVA with IP-10 and IL-10. Additionally, a direct relationship was confirmed between the change in CST and VCAM-1 (Table 4).

**Table 4:** Mediation analysis.

	Indirect		Direct	Total
	Estimate (95%CI) [*P* value]		Estimate (95%CI) [*P* value]	Estimate (95%CI) [*P* value]
Changes in BCVA	Baseline BCVA	Change in CST		
Ang-1	0.0057 (-0.032–0.043) [0.77]	0.023 (-0.057–0.10) [0.58]	0.31 (-0.145–0.65) [0.061]	0.34 (3.7*10 ^**− 4**^ **–0.69) [< 0.05]**
IP-10	−0.039 (-0.099–0.019) [0.19]	−0.014 (-0.047–0.091) [0.41]	**0.15 (0.041–0.27) [0.008]**	0.10 (-0.013–0.21) [0.084]
IL-10	−0.033 (-0.093–0.026) [0.27]	−0.0018 (-0.023–0.020) [0.87]	**0.13 (0.0071–0.26) [0.038]**	0.097 (-0.027–0.22) [0.13]
GM-CSF	−0.042 (-0.12–0.033) [0.27]	0.0022 (-0.020–0.025) [0.84]	0.14 (-0.13–0.30) [0.072]	0.10 (-0.046–0.25) [0.18]
CXCL-13	−0.048 (-0.14–0.046) [0.31]	0.0011 (-0.031–0.033) [0.95]	0.17 (-0.031–0.38) [0.097]	0.13 (-0.073–0.32) [0.22]
**Change in CST**	**Baseline CST**			
VCAM-1	−25.5 (-92–41) [0.45]		**58.6 (1.6–116) [0.044]**	33.0 (-56–122) [0.46]
Galectin-1	−22.6 (-81–36) [0.45]		27.0 (-26–80) [0.32]	4.36 (-75–84) [0.91]
P-Selectin	−20.2 (-65–25) [0.38]		−16.5 (-59–26) [0.45]	−36.7 (-99–25) [0.24]

BCVA, best corrected visual acuity; CST, central subfield retinal thickness; CCT, central choroidal thickness.

## Discussion

In the current study, we included 34 eyes of 32 patients who continued treatment for 1 year. As expected, BCVA significantly improved with a significant reduction in CST and CCT, and the injection interval at the last visit was over 12 weeks in over 90% of eyes. Neither baseline clinical features, including pachychoroid and drusen features, nor the response during the induction phase were associated with changes in BCVA, CST, and CCT or the injection interval at the last visit. VEGF-A was significantly higher in participants with a dry macula at week 16. IL-10, IFN-γ, GM-CSF, and P-selectin were significantly higher in patients with a 16-week injection interval compared with those with 12- or 8-week intervals. Higher levels of galectin-1 and VCAM-1 and a lower level of P-selectin were significantly associated with better changes in CST. Ang-1, IP-10, IL-10, and GM-CSF were significantly associated with better changes in BCVA. Mediation analysis revealed that Ang-1, IP-10, and IL-10 were significantly associated with the change in BCVA, independent of the baseline BCVA or change in CST.

### Clinical outcomes

Overall, the current results are consistent with the results of the pivotal phase III TENAYA and LUCERNE studies, similar to the previously reported real-world clinical outcomes^1,2,12,30,31^. Presumably, the injection interval at the last visit in the present study was better because of the flexibility of the current regimen^32^. The modified treat-and-extend regimen of the current study tolerated subretinal fluid less than 100 μm in height and did not employ changes in BCVA as a disease activity criteria. The current disease activity criteria were based on the FLUID study and post-hoc analyses of the other clinical trials, which reported that strict fluid control may not necessarily be associated with better visual acuity maintenance^8–11^. It is now generally agreed that treatment-resistant subretinal fluid may be tolerable when treating nAMD patients with currently available anti-VEGF drugs^33,34^. We believe that careful monitoring is needed to assess how allowing subretinal fluid impacts long-term visual outcomes when using longer-durability drugs such as faricimab, especially when the treatment intervals become extended over 16 weeks.

### Anatomical outcomes and cytokines

From an anatomical perspective, the current study suggests that faricimab is effective for VEGF-driven nAMD in the loading phase, with multiple cytokines associated with vascular instability or fibrosis appearing to contribute to its durability. First, as expected, a significant association was found between a dry macula at week 16 and an elevated aqueous VEGF level, whereas Ang-2 showed no such association. The early treatment response has been reported to correlate with the subsequent treatment response and prognosis^12–14,35^. In the present study, patients with a dry macula at 16 weeks received fewer injections than those with a wet macula (data not shown). Interestingly, a correlation was observed between the change in visual acuity at 1 year after the first IVF and Ang-1. These results suggest that achieving early suppression of exudation requires inhibiting overexpressed VEGF, and that inhibiting Ang-2 may normalize the Ang-1-Tie-2 pathway, potentially leading to favorable long-term visual acuity outcomes. VEGF plays crucial roles in MNV pathogenesis and fluid accumulation in nAMD; however, other factors have also been implicated in the stability of MNV^7^. Indeed, regarding the factors determining the durability in the maintenance phase, there seems to be complex interplay among cytokines with diverse functions. The results support the idea that VEGF inhibition alone is not sufficient to maintain MNV inactive and stabilized and that factors involved in pericyte loss, endothelial tight junction formation, and chronic inflammation play more important roles in promoting the reactivation of MNV.

Previous study reported that baseline rich Ang-2 level in the aqueous humor was associated with better treatment response to faricimab injection for treatment naïve nAMD^36^. In the present study, we found no association between baseline Ang-2 concentration and treatment response, contrary to previous reports. This discrepancy may be attributed to differences in the assessment timing; the previous study investigated the relationship between Ang-2 levels and the therapeutic effect of the initial administration, whereas our study compared mid- to long-term treatment outcomes. Furthermore, the baseline aqueous humor Ang-2 concentration in our cohort (64.17 ± 73.32 pg/ml) was considerably higher than the mean concentration reported in their study group (6.35 ± 6.39 pg/ml), presumably because of using different analytical platforms. These could be potential reasons for the discrepancies between our current findings and previous reports, necessitating further investigation with a larger cohort.

IL-10, IFN-γ, GM-CSF, and P-selectin were associated with the injection interval, while galectin-1, VCAM-1, and P-selectin were associated with CST changes. Elevated levels of IFN-gamma and other mediators are indicative of a pronounced inflammatory milieu, a condition that generally implies the need for a greater number of injections. Although it sounds rational, this assumption may not hold true based on our current observations. It should also be noted that these factors are not only involved in angiogenesis and inflammation but also fibrosis. For example, galectin-1 and VCAM-1 are reportedly associated with inflammation^37–41^ and are implicated in transforming growth factor-β-driven fibrosis in nAMD^42,43^. In particular, intraocular VCAM-1 levels are significantly higher in nAMD patients with macular fibrosis and positively correlated with CST^44,45^.

Future studies should place more emphasis on factors that control chronic disease activity in nAMD.

### Association between the change in BCVA and Ang-1

Few studies have focused on factors that directly impact visual function, partly due to the outdated belief that visual and anatomical outcomes are closely linked. In this study, after adjustment for each baseline value, age, sex, and axis length, lower levels of Ang-1, IP-10, IL-10, GM-CSF, and CXCL-13 were significantly associated with better changes in BCVA. This partially replicates our prior study showing that CXCL-13 was associated with the changes in BCVA with ranibizumab therapy^46^. Ang-1 was not analyzed in the previous study; however, it is worth noting that a lower level of Ang-1 was associated with better BCVA changes in the current study.

We believe there are two possible mechanisms underlying the effect of Ang-1 on visual function. First, the classical Ang-1/Tie-2 pathway contributes to vessel stability, allowing stabilized choriocapillaris to supply essential nutrients and neurotrophic factors to photoreceptors. Importantly, Ang-1 promotes vascular maturation through the recruitment of pericytes to newly formed capillaries, while Ang-2 acts as an antagonist of Ang-1, destabilizing the mature blood vessels^47^. Second, Ang-1-Tie-2 signaling may have direct neurotrophic functions, as suggested by recent studies^48–50^. Ang-2 acts as a competitive antagonist of Ang-1, inhibiting Tie-2 activation. In an Ang1-rich environment, Ang-2 inhibition may have limited therapeutic efficacy because Ang-1 is already actively engaging the Tie-2 receptor, with minimal competition from Ang-2. However, when Ang-1 levels are low, Ang-2 blockade may have a more pronounced effect. Without Ang-2-mediated inhibition, even small amounts of Ang-1 can activate the Tie-2 receptor, promoting vessel stability and improving photoreceptor function, which explains why a lower level of Ang-1 was associated with better BCVA changes in the current study.

### Other cytokines

There are several interesting cytokines that merit discussion. As a future druggable target, we believe that the ELR-negative CXC ligand IP-10^19,51^ is particularly important. Previous studies showed higher IP-10 in nAMD eyes correlated with greater CST^17–19,52,53^. In the current study, a lower level of IP-10 was significantly associated with a better change in BCVA, not with a change in CST. IP-10 is a specific chemotactic factor for Th1 cells that activates the Th1 cell-mediated immune response by binding to the common C-X-C motif chemokine receptor 3 receptor and is secreted by leukocytes such as monocytes and macrophages and by non-leukocytes such as endothelial cells and smooth muscle cells^54^. IP-10 has antiangiogenic and antifibrotic effects in response to neovascularization^55,56^.

Previous reports showed that IL-10 was higher in eyes with nAMD compared with controls^19,57^ and that eyes with a higher IL-10 level had a better response to anti-VEGF treatment^57^. IL-10 is generally considered an anti-inflammatory cytokine that inhibits inflammation and the synthesis of proinflammatory cytokines^58^. IL-10 regulates the early and late migration of macrophages into and out of the injured nerve, reduces the expression of proinflammatory chemokines and cytokines, and promotes the transition of macrophages from a proinflammatory to an anti-inflammatory phenotype through myelin phagocytosis^59^.

GM-CSF is a growth factor that promotes the activation and survival of microglial cells and macrophages^60^. Activated microglia accumulating in the retina have been implicated in the pathogenesis of nAMD by mediating an inflammatory response^61–63^.

Previous studies showed that the levels of IFN-γ were significantly elevated in patients with AMD and that this inflammatory cytokine induced ferroptosis in RPE cells and aggravated the development of AMD^20,21^.

### Limitations

This study has some limitations. First, all patients were Japanese, and the results might be influenced by regional differences among the Japanese population. Second, the number of eyes classified as each MNV subtype was small, and the efficacy for each MNV subtype was not adequately evaluated. Similarly, the percentage of participants with a greater than 12-week injection interval was higher than in previous studies. This favorable outcome might be due to the small number of eyes and the treatment regimen used in the current study. Moreover, only baseline cytokine levels were quantified and compared between clinical outcomes, while changes in cytokine concentrations over time in response to treatment were not assessed. Further studies are thus needed to evaluate the efficacy of faricimab.

## Conclusion

From an anatomical perspective, faricimab was shown to be effective for VEGF-driven nAMD in the loading phase while multiple cytokines associated with vascular instability or fibrosis appear to contribute to its durability. In particular, Ang-1 may be linked to visual improvement, suggesting that the neurotrophic effects of Ang-1 could be enhanced by Ang-2 inhibition. Further studies are warranted.

## Supplementary Information

Below is the link to the electronic supplementary material.

Supplementary Material 1

Supplementary Material 2

Supplementary Material 3

Supplementary Material 4

Supplementary Material 5

Supplementary Material 6

Supplementary Material 7

Supplementary Material 8

## Data Availability

The datasets generated during and/or analyzed during the current study are available from the corresponding author on reasonable request.

## Abbreviations

Ang: Angiopoietin
BCVA: Best corrected visual acuity
CCL: C-C motif chemokine ligand
CCT: Central choroidal thickness
CST: Central subfield thickness
CXCL: C-X-C motif chemokine ligand
G-CSF: Granulocyte colony-stimulating factor
GM-CSF: Granulocyte macrophage colony-stimulating factor
ICAM: Intercellular adhesion molecule
IFN-γ: Interferon-gamma
IL: Interleukin
IP: Interferon-gamma-inducible protein
IVF: Intravitreal faricimab
MMP: Matrix metallopeptidase
MNV: Macular neovascularization
nAMD: Neovascular age-related macular degeneration
OCT: Optical coherence tomography
PDGF: Platelet-derived growth factor
TNF-α: Tumor necrosis factor-alpha
VCAM: Vascular cell adhesion molecule
VEGF: Vascular endothelial growth factor
