# Supplementary figures and images for: One year outcomes of intravitreal faricimab for treatment Naïve neovascular AMD and associations with baseline aqueous humor cytokines
Source: Sci Rep. 2025 Dec 29;15:44717. doi: 10.1038/s41598-025-28911-9 (PMC12750000; doi:10.1038/s41598-025-28911-9)

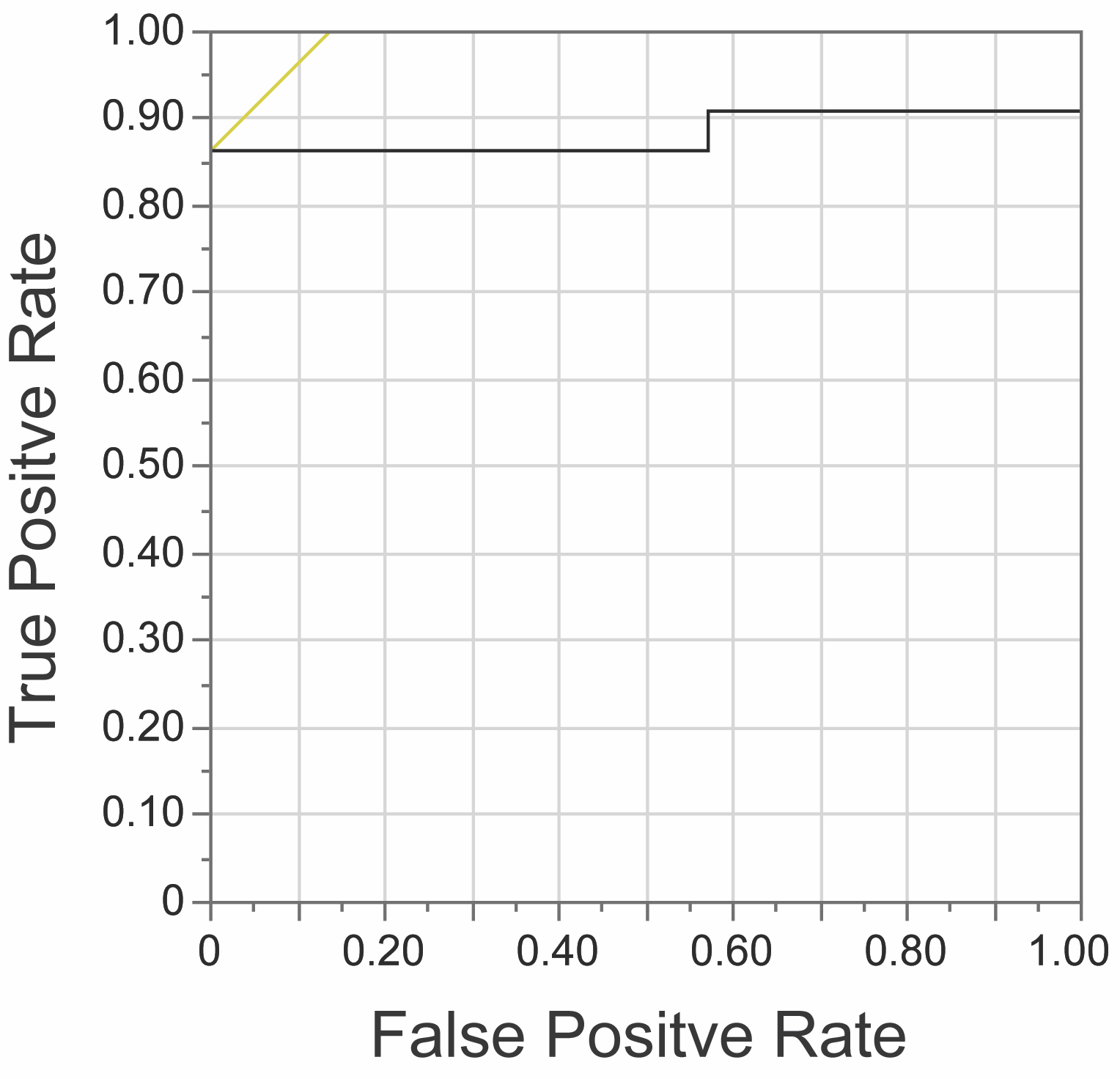

Supplement: Supplementary file 1 — Supplementary Material 1 [file 41598_2025_28911_MOESM1_ESM.tiff]

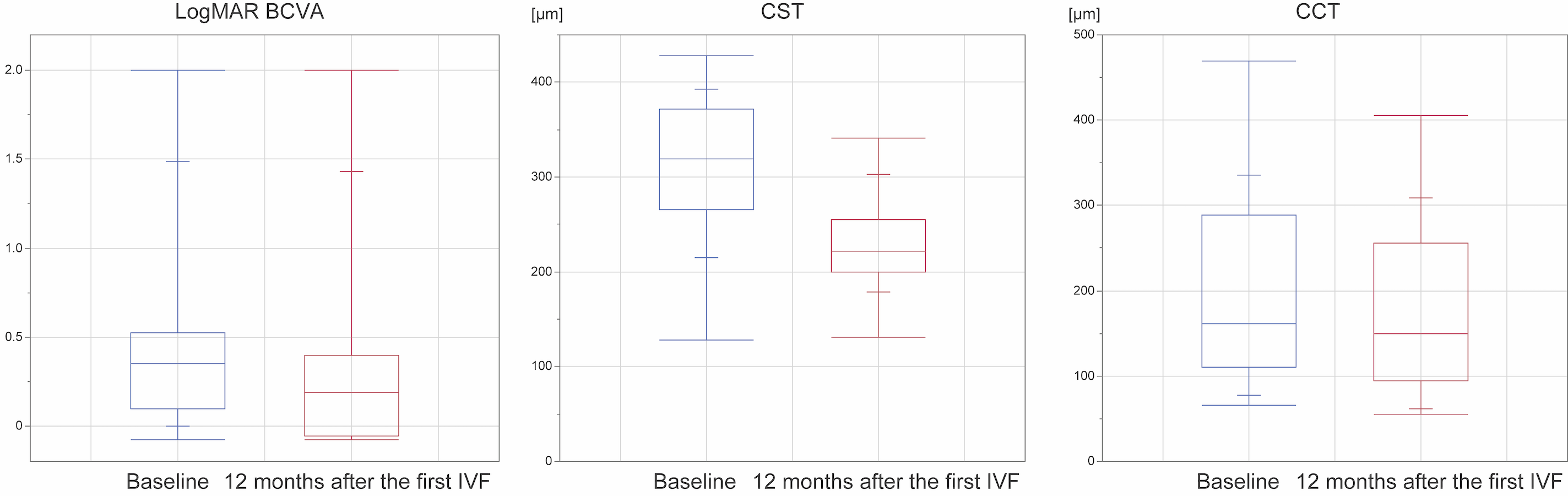

Supplement: Supplementary file 2 — Supplementary Material 2 [file 41598_2025_28911_MOESM2_ESM.tiff]

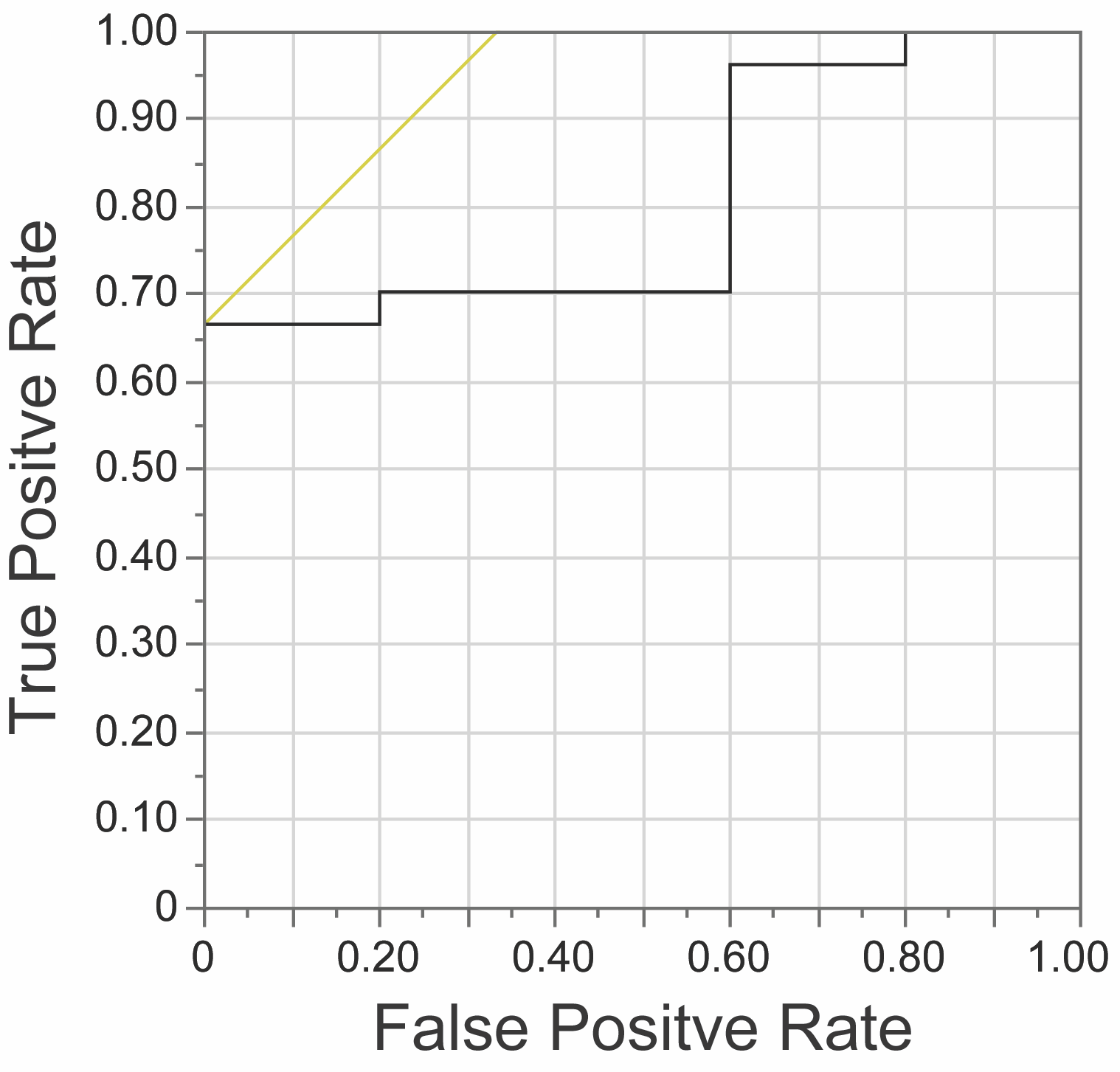

Supplement: Supplementary file 3 — Supplementary Material 3 [file 41598_2025_28911_MOESM3_ESM.tiff]

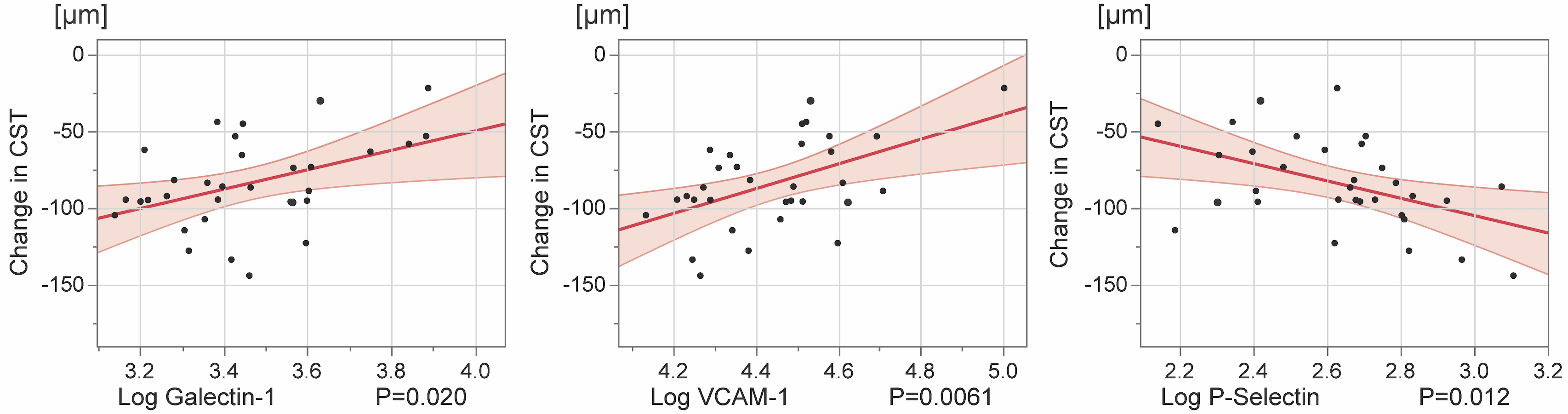

Supplement: Supplementary file 4 — Supplementary Material 4 [file 41598_2025_28911_MOESM4_ESM.tiff]

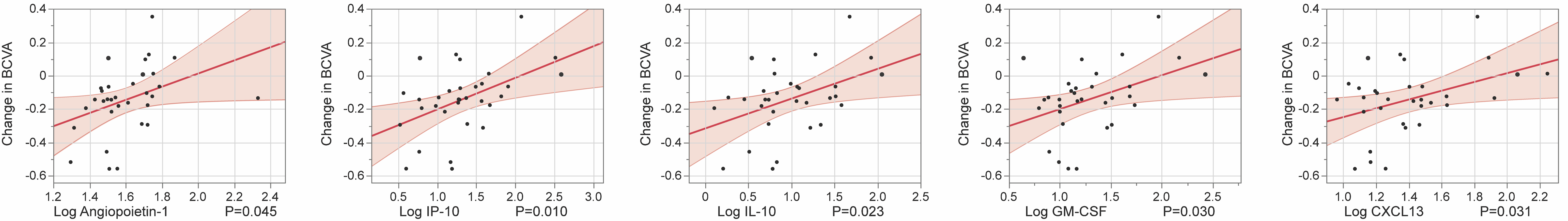

Supplement: Supplementary file 5 — Supplementary Material 5 [file 41598_2025_28911_MOESM5_ESM.tiff]

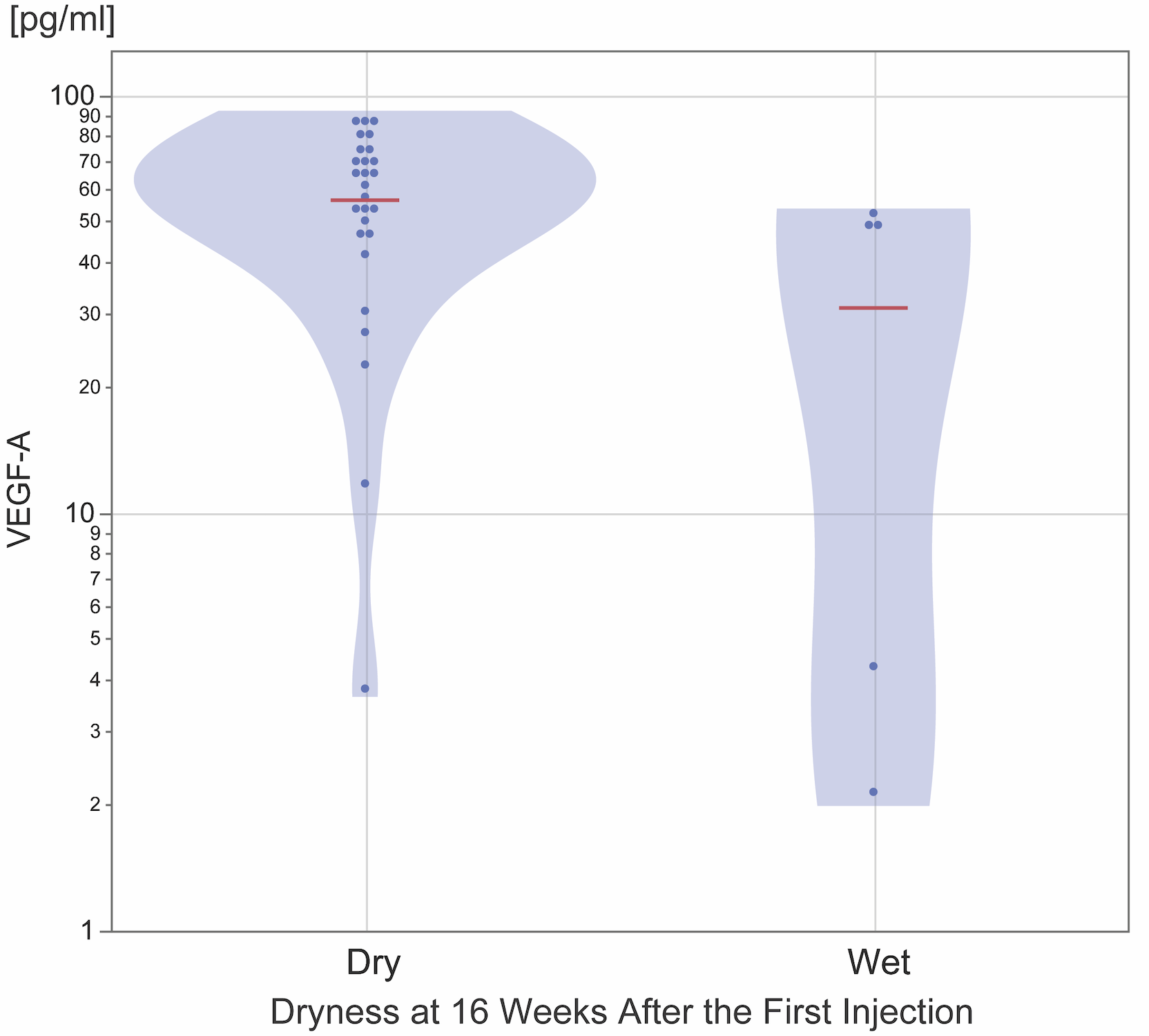

Supplement: Supplementary file 6 — Supplementary Material 6 [file 41598_2025_28911_MOESM6_ESM.tiff]

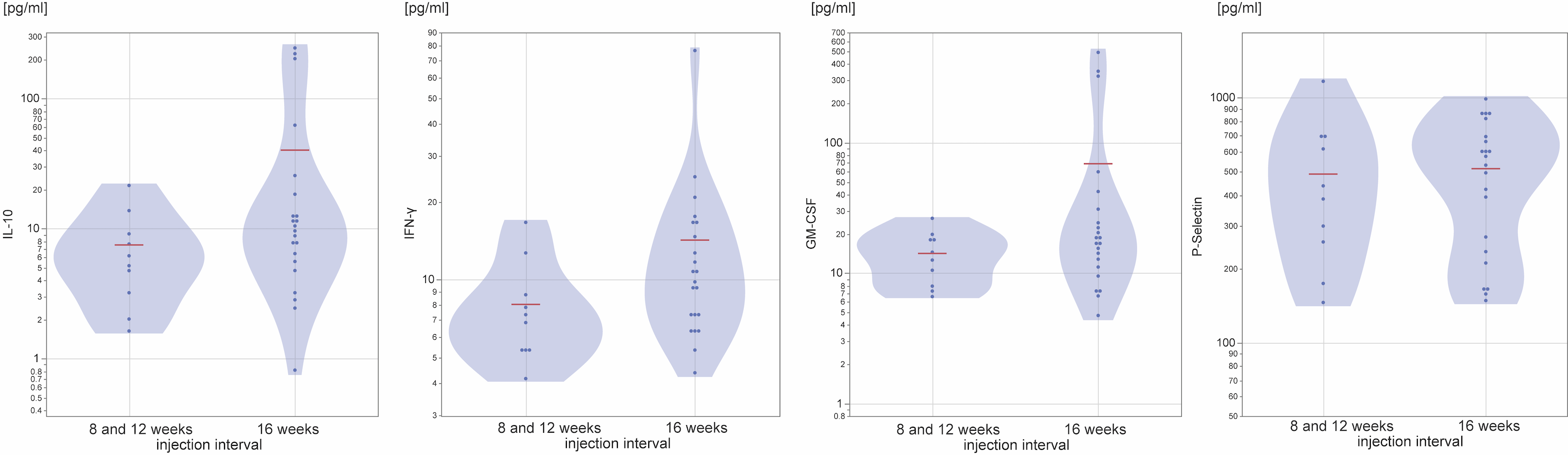

Supplement: Supplementary file 7 — Supplementary Material 7 [file 41598_2025_28911_MOESM7_ESM.tiff]
