# Supplementary material for: One year outcomes of intravitreal faricimab for treatment Naïve neovascular AMD and associations with baseline aqueous humor cytokines
Source: Sci Rep. 2025 Dec 29;15:44717. doi: 10.1038/s41598-025-28911-9 (PMC12750000; doi:10.1038/s41598-025-28911-9)
Supplement: Supplementary file 8 — Supplementary Material 8 [file 41598_2025_28911_MOESM8_ESM.pdf]

Supplementary Information for

One Year Outcomes of Intravitreal Faricimab for Treatment Naïve Neovascular AMD and Associations With Baseline Aqueous Humor Cytokines

Satoru Inoda, MD, PhD [1], Hidenori Takahashi, MD, PhD [1, 2], Yuto Hashimoto, MD[1], Hana Yoshida, MD[1], Hironori Takahashi, MD[1], Takuya Takayama, MD[1], Shouma Tsuchiya, MD[1], Daizo Matsumoto, MD[1], Hidetoshi Kawashima, MD, PhD[1], Toshikatsu Kaburaki, MD, PhD[1], Yasuo Yanagi, MD, PhD[3, 4]

[1] Department of Ophthalmology, Jichi Medical University, Shimotsuke-shi, Tochigi, Japan
[2] Center for Cyber Medicine Research, University of Tsukuba, Tsukuba-shi, Ibaraki, Japan
[3] Department of Ophthalmology and Micro-Technology, Yokohama City University, Yokohama, Japan
[4] Retina Research Group, Singapore Eye Research Institute, Singapore Eye-ACP, Duke-NUS Medical School, National University of Singapore, Singapore

Contents

Supplementary Table S1

Supplementary Figure Legends S1~7

Supplementary Table S1. Association of Cytokines and Changes in BCVA and CST After Adjustment for Baseline Values, Age, Sex, and Axial Length with Bootstrap Analysis (1000 Resamples)

	Changes in BCVA		Changes in CST	
	**Estimate (95% CI)**	***P* value**	**Estimate (95% CI)**	***P* value**
**Angiopoietin-1**	0.39 (0.071 – 1.2)	**0.045**		
**IP-10**	0.18 (0.032 – 0.34)	**0.010**		
**IL-10**	0.18 (0.026 – 0.33)	**0.023**		
**GM-CSF**	0.20 (-0.038 – 0.46)	**0.030**		
**VCAM-1**			81 (15 – $1.3*10^2$)	**0.0061**
**CXCL-13**	0.26 (0.028 – 0.54)	**0.031**		
**Galectin-1**			63 (4.7 – $1.0*10^2$)	**0.020**
**P-selectin**			-57 ($-1.1*10^2$ – -7.1)	**0.012**

BCVA, best corrected visual acuity; CST, central subfield retinal thickness; IVF, intravitreal faricimab; CI, confidence interval

Supplementary Figure Legends

Supplementary Fig. 1 Changes in BCVA, CST and CCT.
Best corrected visual acuity (BCVA), central retinal subfield thickness (CST) and central choroidal thickness (CCT) 12 months after the first intravitreal faricimab injection (IVF) were significantly improved. (all $P < 0.001$)

Supplementary Fig. 2 Association between VEGF-A and macular dryness at 16 weeks after the first IVF.
VEGF-A was significantly higher in patients with a dry macula at 16 weeks post-initial intravitreal faricimab injection (IVF).

Supplementary Fig. 3 ROC curve for Change in BCVA
Area under the receiver operating characteristic curve analysis showed area under the curves was 0.81 with a sensitivity of 67% and specificity of 100%.

Supplementary Fig. 4 Association between cytokines and injection interval 12 months after the first IVF.
At 1 year post-initial intravitreal faricimab injection (IVF), IL-10, IFN-γ, GM-CSF, and P-selectin were significantly elevated in patients with 16-week injection intervals compared to those with 12- or 8-week intervals. (P = 0.0015, 0.031, 0.014, and 0.019)

Supplementary Fig. 5 ROC curve for Injection Interval one year after the first IVF.
Area under the receiver operating characteristic curve analysis showed area under the curves was 0.88 with a sensitivity of 86% and specificity of 100%.

Supplementary Fig. 6 Association between cytokines and change in CST 12 months after the first IVF.
Higher levels of galectin-1 and VCAM-1 and a lower level of P-selectin were significantly associated with a greater reduction in central retinal subfield thickness (CST) after adjusting baseline CST, age,

sex and axial length. (*P* = 0.020, 0.0061, and 0.012)

Supplementary Fig. 7 Association between cytokines and change in BCVA 12 months after the first IVF.

As a functional outcome, we analyzed factors associated with changes in best corrected visual acuity (BCVA), which revealed that lower levels of Ang-1, IP-10, IL-10, GM-CSF, and CXCL-13 were significantly associated with better changes in BCVA after adjusting baseline BCVA, age, sex and axial length. (*P* = 0.045, 0.010. 0.023 0.030, and 0.031)
